# A Swine Model of Traumatic Brain Injury: Effects of Neuronally Generated Electromagnetic Fields and Electromagnetic Field Stimulation on Traumatic Brain Injury-Related Changes

**DOI:** 10.7759/cureus.42544

**Published:** 2023-07-27

**Authors:** James Brazdzionis, Mohamed M Radwan, Finosh Thankam, Merlin Rajesh Lal, David Baron, David A Connett, Devendra K Agrawal, Dan E Miulli

**Affiliations:** 1 Neurosurgery, Riverside University Health System Medical Center, Moreno Valley, USA; 2 Translational Research, College of the Osteopathic Medicine of the Pacific, Western University of Health Sciences, Pomona, USA; 3 Psychiatry and Behavioral Sciences, College of the Osteopathic Medicine of the Pacific, Western University of Health Sciences, Pomona, USA

**Keywords:** electromagnetic field, traumatic brain injury, sensors, swine model, tbi, electromagnetic field stimulation, stimulation, controlled cortical impact, emf, neuronal circuit

## Abstract

Background and objective

Traumatic brain injury (TBI) has been associated with aberrations in neural circuitry attributable to the pathology resulting in electromagnetic field (EMF) changes. These changes have been evaluated in a variety of settings including through novel induction sensors with an ultra-portable shielded helmet and EMF channels with differences identified by comparing pre-injury and post-injury states. Modulation of the EMF has undergone cursory evaluation in neurologic conditions but has not yet been fully evaluated for clinical effects in treatment. Target EMF stimulation using EMF-related changes preoperatively to postoperatively has not yet been attempted and has not been completed using induction sensor technology. Our objectives in this study were twofold: we wanted to test the hypothesis that targeted stimulation using an EMF signal generator and stimulator to abnormal thresholds identified by real-time measurement of EMFs may enable early resolution of EMF changes and treatment of the TBI as modeled through controlled cortical impact (CCI); we also aimed to assess the feasibility of attempting this using real-time measurements with an EMF shielded helmet with EMF channels and non-contact, non-invasive induction sensors with attached EMF transmitters in real-time.

Methods

A singular Yucatan miniswine was obtained and baseline EMF recordings were obtained. A CCI of TBI and postoperative assessment of cortical EMF in a non-invasive, non-contact fashion were completed. Alterations in EMF were evaluated and EMF stimulation using those abnormal frequencies was completed using multiple treatments involving three minutes of EMF stimulation at abnormal frequencies. Stimulation thresholds of 2.5 Hz, 3.5 Hz, and 5.5 Hz with 1 V signal intensity were evaluated using sinusoidal waves. Additionally, stimulation thresholds using differing offsets to the sine wave at -500 mV, 0 mV, and 500 mv were assessed. Daily EMF and post-stimulation EMF measurements were recorded. EMF patterns were then assessed using an artificial intelligence (AI) model.

Results

AI modeling appropriately identified differences in EMF signal in pre-injury, post-injury, and post-stimulation states. EMF stimulation using a positive offset of 500 mV appeared to have maximal beneficial effects in return to baseline. Similarly targeted stimulation using thresholds of 2.5 Hz and 5.5 Hz with a positive 500 mV offset at 1 V allowed for recovery of EMF patterns post-injury towards patterns seen in baseline EMF measurements on stimulation day seven (postoperative day 17).

Conclusion

Stimulation of neural circuits with targeted EMF in a sinusoidal pattern with targeted thresholds after measurement with induction sensors with shielding isolated to a Mu-metal and copper mesh helmet and EMF channels is efficacious in promoting neuronal circuit recovery to preoperative baselines in the TBI miniswine model. Similarly, our findings confirm the appropriateness of this translational model in the evaluation of brain neuronal circuit EMF and that preoperative and post-trauma differences can be appropriately assessed with this technology.

## Introduction

Traumatic brain injury (TBI) is a disabling condition frequently associated with deaths worldwide; secondary injury occurs from associated processes involving apoptosis, inflammation, and disruption of signaling, resulting in the worsening of the initial insult and continued injury [1-3]. Treatments of TBI have focused on improving the environment of recovery to limit secondary injury to promote healing and recovery [4].

It has been found that TBI causes disruptions in neuronal pathways and signaling [1,5,6]. These changes have been measured in various ways biochemically, through imaging changes in MRI and functional MRI and in electrophysiologic monitoring such as magnetoencephalography (MEG) and electroencephalography (EEG) [6-8]. Further assessment has been conducted in swine models, which have evaluated the electromagnetic field (EMF) in real-time using a shielded helmet and proprietary induction-type EMF sensors in swine models, and has identified physiologic changes in cortical signaling after controlled cortical impact (CCI) [9].

Additionally, early studies have investigated transcranial magnetic stimulation (TMS) on TBI with regression of injury in histology and physiologic improvements [10,11]. Additionally, EMF modulation has undergone early investigation in alternative pathologies such as stroke, Alzheimer’s disease, and spinal cord injury [12-14]. These studies, however, did not measure changes in EMF prior to the guidance of stimulation thresholds. Clinically, measurement of EMF and modulation has been limited due to technical and cost barriers. Some limitations include the requirement of supercooling technologies for MEG measurements, shielded rooms to exclude external EMF, and high costs [15,16]. To combat this, technology has been developed to isolate and limit the shielding to smaller configurations. It has been found that shielding confined to a helmet using novel induction sensors and EMF channels containing the sensor has been sufficient to evaluate neuronal activity in swine and in human subjects [9,17-21].

To address these challenges, technological development is ongoing to limit the need for such extensive shielding. It has been discovered that helmets in conjunction with novel induction sensors and EMF channels are sufficient to measure cortical EMF activity in swine and human subjects [9,21]. This helmet-based solution is more affordable, portable, and can be used continuously while effectively excluding unwanted EMF "noise". To our knowledge, brain neuronal circuit stimulation technologies have not yet been investigated using targeted EMF measurements developed from pre-CCI to post-CCI changes to attempt to directly stimulate abnormal pathways and modulate the abnormal EMF signal. It is thought that modulating the abnormal EMF signal may reduce deleterious effects from inflammatory cytokines, abnormal signaling, and cortical depressions and may produce regenerative effects on neurons to allow for the recovery of these injured cells and circuits [2,13,22,23].

We hypothesized that targeted stimulation using an EMF signal generator and stimulator to abnormal thresholds identified by real-time measurement of EMFs may allow for early resolution of EMF changes and treatment of TBI as modeled through CCI. We also aimed to assess the feasibility of attempting this using real-time measurements with an EMF-shielded helmet with EMF channels and non-contact, non-invasive induction sensors with attached EMF transmitters in real-time. These sensors and helmet construct have been utilized in a swine model with Yucatan minipigs and demonstrated efficacy in measuring and, importantly, discerning swine cortical EMF and preoperative changes from postoperative ones [9].

## Materials and methods

This study was reviewed and granted approval by the Western University of Health Sciences Institutional Animal Care and Use Committee under protocol number R23IACUC003. A single swine model of TBI was utilized to evaluate the effects of TBI and EMF stimulation on the EMF generated by cortical neurons as a pilot trial. Yucatan minipigs were obtained from Premier BioSource (Romona, CA) and were cared for in a vivarium. Preoperative measurements of the EMF were performed daily on a Yucatan minipig through the protocol documented by Brazdzionis et al. by using proprietary induction sensors (model BS-1000, Quasar Federal Systems, San Diego, CA) and dual-layered Mu-metal (MuMETAL®, Magnetic Shield Corporation, Bensenville, IL) [9]. Sensors were placed in the left frontal region (sensor B319), right frontal region (sensor By), left parietal region (Sensor Bx), and right parietal region (sensor Bz) after guiding them through EMF channels into the shielded helmet constructed with dual-layered Mu-metal as inner and outer layers with a 2.5-cm air gap and inner and outer interlaced copper mesh protected within interior and exterior plastic layers [9]. Of note, the sensor sensitivity is 1pT/rtHz.

Data were captured using a National Instruments data card (16-bit, National Instruments Corporation, Austin, TX). A low-pass filter was used for dynamic filtering with filtering set at 2 kHz. The sample capture rate was 5 kilo samples per second. Data were analyzed using Igor® Pro version 8 (WaveMetrics, Lake Oswego, OR). Analyzed data utilized a fast Fourier transform (FFT) algorithm to analyze the frequency domain to effectively measure the summative amplitudes of measured EMF at a desired frequency within a measured 20-second bin allowing for the evaluation of 1000,000 data points per Hz. During data recordings, consistent with previous studies, a 20-second bin was selected to be evaluated wherein the pig was ensured to have minimal movement, and the helmet was located precisely over the swine’s head with sensors oriented in the appropriate locations as above. Similar to previous studies, licorice was used to direct the attention of the subject within the corner of a pen for data collection [9]. Baseline room EMF noise data were measured in the vivarium using the sensor, helmet, and channel setup used on the swine, albeit without any subject within the helmet to evaluate just the room data.

Traumatic brain injury induction

After baseline measurements were obtained daily for seven measurements, the Yucatan minipig was taken to the OR to undergo CCI. Induction of TBI through CCI was completed with the same protocol as documented by Brazdzionis et al., through the usage of a small craniotomy with the site guided by bregma and the use of an 8.359-g stainless steel ball for an impact force dropped through a galvanized steel tube to fall at a rate of 3.579 m/second [9]. EMF was measured preoperatively while in the operating suite while under general anesthesia and then measured post-injury while under anesthesia. The anesthetics utilized were consistent with those used in previous studies: acepromazine, ketamine, Telazol, xylazine, propofol, and isoflurane [9]. Swine vitals were recorded and monitored during the surgery in the pre-induction, post-induction, pre-injury, and post-injury phases. Blood samples were obtained to collect serum of the subject on the day of surgery, postoperative day four, postoperative day seven, and on the day of sacrifice for future studies.

After induction of the TBI through CCI, the subject was brought back to its pen within the vivarium for recovery. Once it was ambulatory and able to tolerate oral intake, postoperative EMF measurements were taken. The swine was then monitored through postoperative day two with daily EMF measurements. The postoperative day two EMF measurements were then evaluated and targeted EMF stimulation was started.

Electromagnetic field stimulation in swine subjects

On postoperative day two, a protocol was utilized wherein baseline EMF measurements were taken of the room and the swine model. This was followed by initial EMF stimulation and then followed by measurements of the pig’s cortically generated EMF five minutes post-stimulation using an EMF signal generator.

Starting with these stimulation trials and continuing through the remainder of the study, stimulation occurred in three-minute intervals. Stimulation was directed through sensor B319 unless otherwise specified, which was oriented in the left frontal EMF channel traversing through the helmet and directed at the region of induced TBI. The helmet containing the sensor was oriented just above the scalp of the pig oriented consistently with sensor B319 over the region of injury in the left frontal region, sensor Bx in the left parietal region, sensor By in the right frontal region, and sensor Bz in the right parietal region, and the snout of the minipig was ensured to be maintained continuously forward. Licorice was fed to the minipig and was utilized as a distraction for the pig to keep the swine from changing position during the trial measurement and stimulation trials. To complete the stimulation, an EMF signal generator was utilized with differing settings throughout the trial, which would be defined. These initial stimulation trials were done to assess the effects of EMF stimulation to determine potential settings for the signal generator to better target alterations in EMF to attempt to treat the changes noted post-TBI. Initial EMF stimulation thresholds on postoperative days two and three were 2.5 Hz and 3.5 Hz with an amplitude of 1 V with no offset using a sinusoidal wave pattern.

After each EMF stimulation trial, post-stimulation EMF measurements were taken five minutes post-stimulation. The EMF measurements were assessed daily for recovery to preoperative baselines. Additionally, during the stimulation trials, baseline room noise data was recorded, as was noise data with the stimulator running using sensors Bx, By, and Bz, measuring without a subject within the helmet.

After the initial two days of EMF stimulation, the stimulation threshold was assessed to evaluate the effects of offsetting the EMF signal generated to shift the sine wave’s midline above or below the zero point. Continuing to use settings of 2.5 Hz and 3.5 Hz with a signal amplitude of 1 V, the offset was set to 500 mV, 0 V, and -500 mV for trials with the evaluation of post-stimulation EMF to assess the effects of offset on cortical activity by reviewing post-stimulation EMF measurements. After a review of these results, it was decided at that point to utilize a standard offset of +500 mV with a setting of 2.5 Hz. Using these now standard settings, on postoperative day seven, with +500 mV offset, 2.5Hz, and 1V settings, an evaluation of stimulation using all sensors simultaneously was completed with a post-stimulation EMF assessment and recorded.

The remaining days of the trial consisted of pre-stimulation measurements, three minutes of stimulation, followed by post-stimulation measurements, using sensor B319 as the stimulating lead during stimulation. Settings of the EMF signal generator continued to be set at +500 mV offset, 2.5Hz, and 1 V. Daily evaluation of the EMF signals was performed and after the assessment, it was noted that at 5.5 Hz, the baseline peak had not returned to baseline by postoperative day 16 while other peaks and valleys had noted some recovery. Therefore, it was decided to shift the signal generation setting to +500 mV, 5.5 Hz, and 1 V for the remainder of the trial with the last day of stimulation and measurements falling on postoperative day 21. After postoperative day 21, the pig was euthanized with serum, and histologic specimens were obtained for future studies. Tissue was obtained and processed within 30 minutes of euthanasia.

Daily post-process measurements of EMFs were assessed independently by two authors (DM and JB) and evaluated for the prominent peaks and valleys in sensor B319 as sensor B319 was oriented over the lesion. These prominent peaks were defined as the inflection point on the graph of maximal amplitude reached wherein the y-value is larger than both of its immediate neighboring points. Valleys were similarly defined as inflection points wherein the amplitude was lower than the values of its immediate neighboring points. After recording these points, patterns were assessed using an artificial intelligence (AI) large language module (LLM) model to assess the preoperative, postoperative, and post-stimulation data sets. Within the post-stimulation data sets trial, the daily baseline data sets taken prior to the stimulation trial of the day were assessed to evaluate the durable effects of stimulation.

## Results

**Figure 1:**
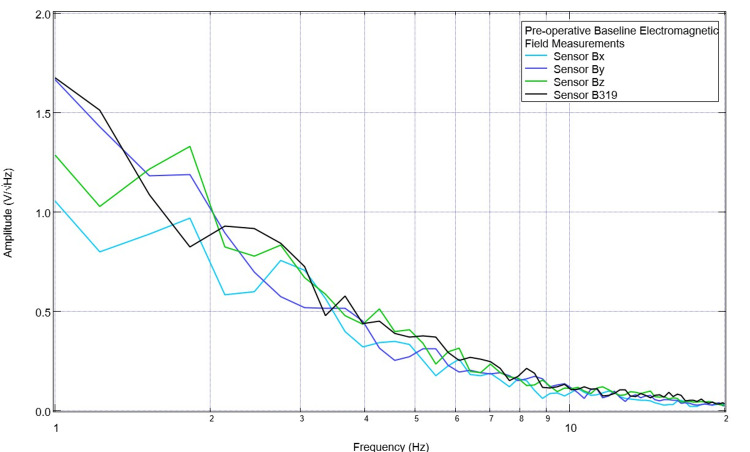
Baseline pre-injury electromagnetic field measurements Baseline electromagnetic field activity was measured using the helmet and sensor construct with a swine subject. A prominent peak near 2.5 Hz and 3.5 Hz was noted. Sensor orientation within the helmet was as follows: B319 in the left frontal region, Bz in the left parietal, By in the right frontal, and Bz in the right parietal

**Figure 2:**
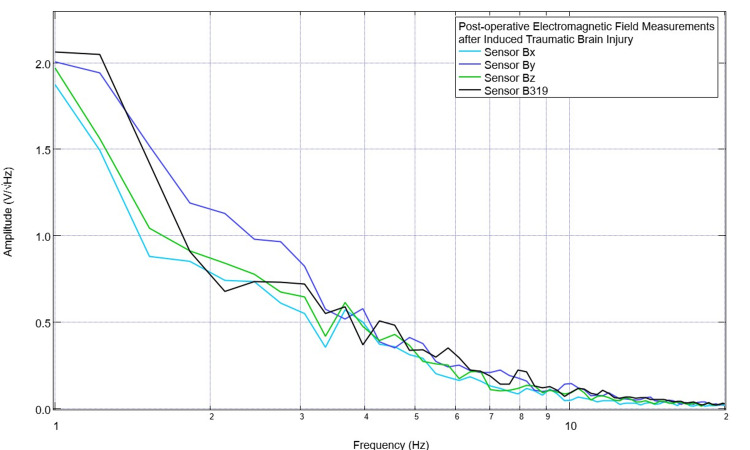
Post-controlled cortical impact electromagnetic field measurements Post-controlled cortical impact electromagnetic field measurements of the swine subject were recorded. Altered morphologic characteristics within the waves compared to pre-injury measurements were noted

The pig model was evaluated daily within the vivarium using the sensors, helmet, and EMF channel to obtain daily baseline EMF measurements. An example of this preoperative initial EMF measurement is presented in Figure 1. This was recorded daily for eight measurements to obtain a subset of baseline EMF to attempt to identify patterns within the preoperative measurements with regard to identified peaks and valleys. Subsequently, the Yucatan minipig underwent CCI surgical intervention, and postoperative EMF measurements were obtained on the day of surgery and for two postoperative days. Similarly, these EMF recordings were assessed for peaks and valleys. An example of this recording is shown in Figure 2. It was noted that preoperatively there were peaks centered around 2.5 +/- 0.3 Hz in six of the seven measurements and 3.5 +/- 0.8 Hz in six of the seven measurements. In the postoperative assessment, the morphology of the waves at these frequencies was noted to be flatter with more of a plateau-type pattern as well as commonly associated with a valley within 0.3 Hz of the measurement; therefore, the values of 2.5 Hz and 3.5 Hz were selected as the programmed frequencies of the EMF function generator in the initial assessment of the effects of stimulation.

**Figure 3:**
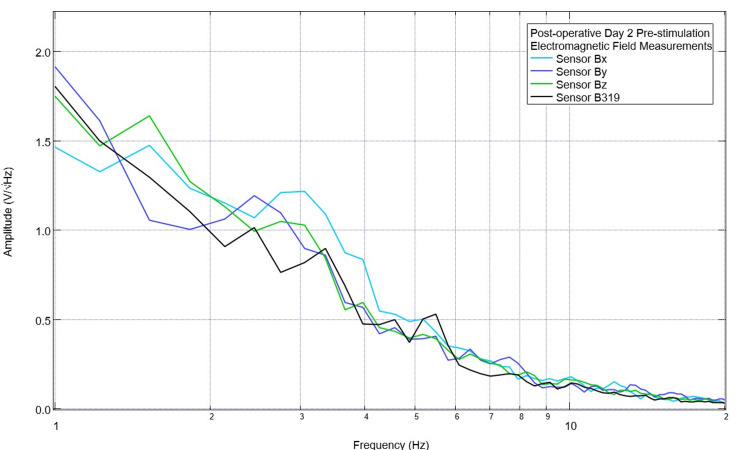
Pre-stimulation electromagnetic field measurements obtained on postoperative day 2 Pre-intervention measurements of the electromagnetic field were noted for the swine subject

**Figure 4:**
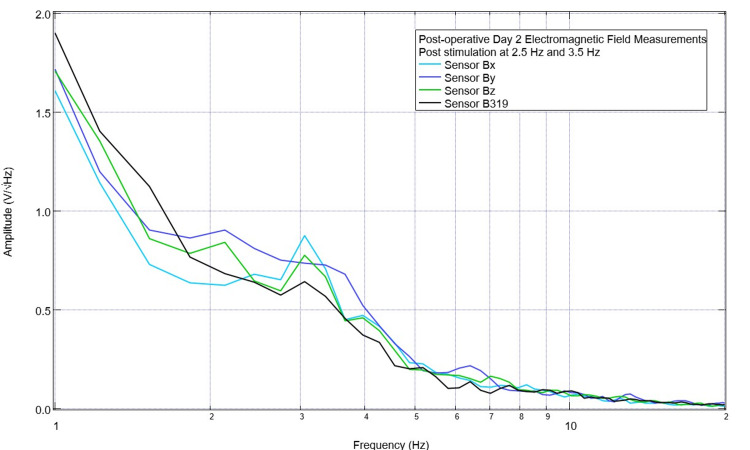
Post-stimulation electromagnetic field measurements on postoperative day 2 Measured electromagnetic fields for the swine subject are seen. Stimulation was completed for three-minute treatments each at 2.5 Hz and 3.5 Hz with no offset set at 1 V

Prior to each stimulation session, the Yucatan minipig underwent a pre-stimulation assessment of the baseline cortical circuit-generated EMF. An example of this baseline assessment is shown in Figure 3. There was a peak noted at 2.5 Hz and 3.5 Hz in sensor B319, but these were corresponding to valleys in sensors Bx and Bz (with a valley at 2.5 Hz and one occurring just after 3.5 Hz). The swine model underwent stimulation at 2.5 and 3.5 Hz using the protocol of three minutes of stimulation using sensor B319 as the stimulating sensor. After stimulation, measurements of the EMF generated by the subject were obtained and are seen in Figure 4. It was noted that the peaks did not reappear at 2.5 Hz and 3.5 Hz post-stimulation but the morphologic patterns of the waves were much different post-stimulation compared to pre-stimulation with the first real peak post-stimulation in sensor B319 occurring at 3.1 Hz. Additionally, the waveform also appeared to have fewer peaks immediately post-stimulation compared to pre-stimulation.

**Figure 5:**
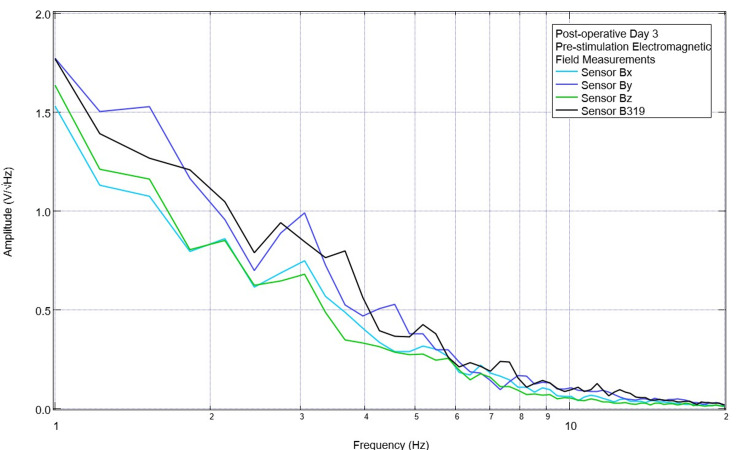
Pre-stimulation baseline electromagnetic field recording obtained on postoperative day 3 Electromagnetic field measurements prior to stimulation on postoperative day 3 are plotted

**Figure 6:**
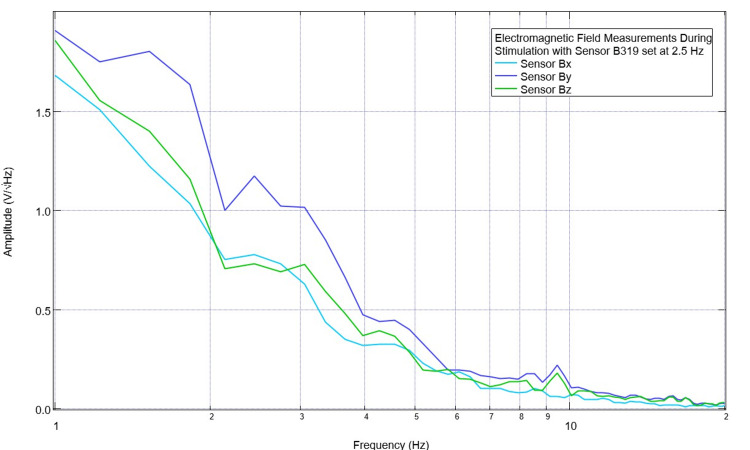
Measurements of the electromagnetic field during stimulation at 2.5 Hz with no offset at 1V on postoperative day 3 Measurements of the swine cortical electromagnetic field were obtained during stimulation on postoperative day 3 using stimulation at 2.5 Hz without an offset set at 1V. Sensor B319 was not recorded as it was utilized for stimulation

**Figure 7:**
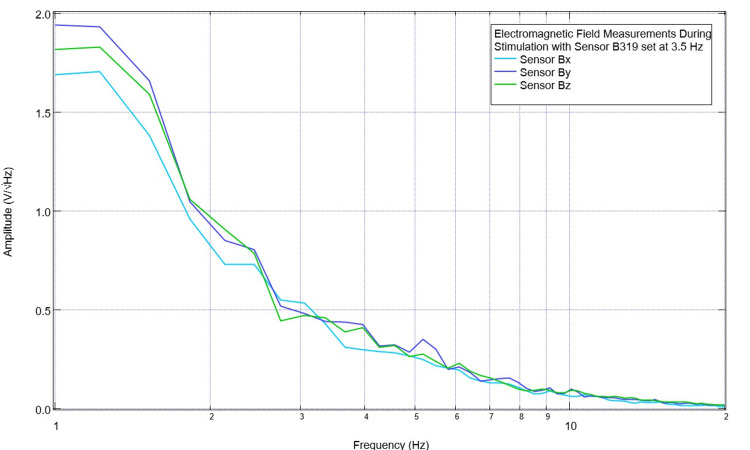
Electromagnetic field recordings during stimulation with 3.5 Hz with no offset at 1V on postoperative day 3 Electromagnetic field recordings were identified occurring during a stimulation trial with the stimulator set at 3.5 Hz with no offset set at 1V. Sensor B319 was not recorded as it was utilized for stimulation

On postoperative day three, continued trials with stimulation at 2.5 Hz and 3.5 Hz were planned. The pre-stimulation baseline EMF waveform is seen below in Figure 5. While earlier there was a large valley seen at 2.5 Hz in all sensors at 3.7 Hz near the previous targeted stimulation, there was now a peak in sensor B319. Sensors Bx, By, and Bz, which were not over the CCI lesion, did not display this peak. On postoperative day three, during stimulation trials, simultaneous recordings were taken of the pig using the sensors not used for stimulation. These recordings are seen in Figures 6-7. It was noted that during stimulation at 2.5 Hz, there was an obvious peak seen in sensors Bx, By, and Bz (most prominent in By directed at the right frontal region) at 2.5 Hz. When stimulating at 3.5 Hz, there was a small peak noted at 3.5 in the Bz sensor (directed at the right parietal region). Additionally, in sensors Bx and By, although the slope continued to be negative at 3.5 Hz, it was less negative than prior to stimulation with a flatter waveform morphology identifying a change in amplitude trending towards but not yet reaching a positive-type morphology.

**Figure 8:**
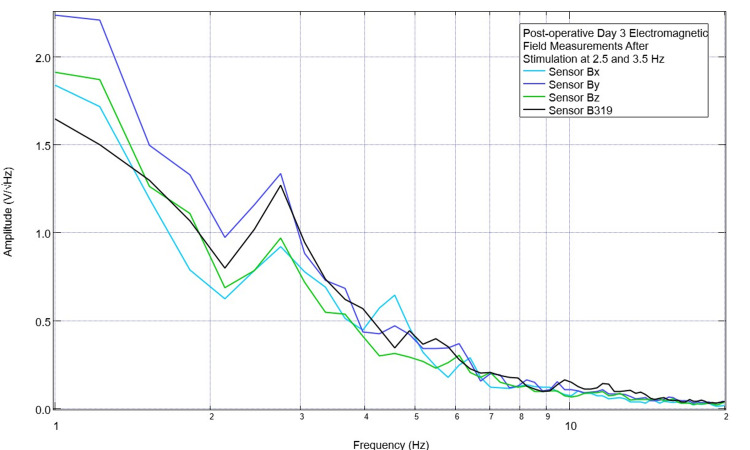
Post-stimulation electromagnetic field recordings on postoperative day 3 after stimulation at 2.5 Hz and 3.5 Hz No offset was utilized in this trial with the stimulator set at 1 V

Post-stimulation assessment of the subject was performed five minutes after stimulation at 2.5 Hz and 3.5 Hz. A graph of this is shown in Figure 8. It was noted that a peak near 2.5 Hz persisted (seen within 0.2Hz of 2.5Hz) and at 3.5 Hz, the morphology of the waveforms for sensors Bx, By, and Bz was less negative than seen at baseline; the peak at 3.5 Hz originally seen in sensor B319 was not present.

**Figure 9:**
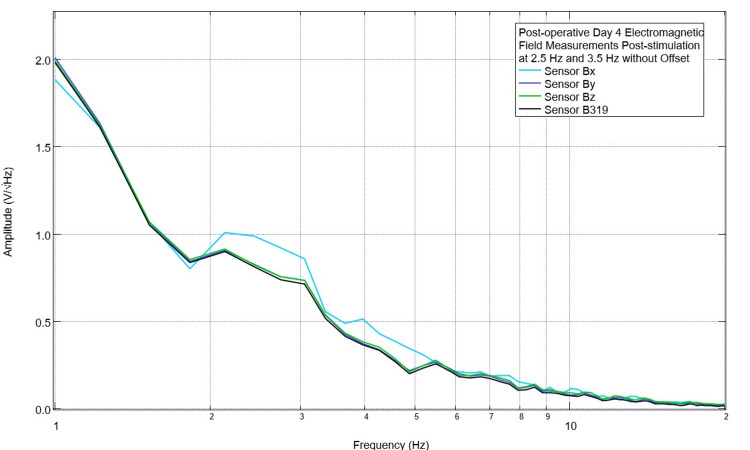
Electromagnetic field measurement from postoperative day 4 after stimulating at 2.5 Hz and 3.5 Hz without offset Recordings were obtained on the swine subject on postoperative day 4 after two stimulation trials with sensor B319 set at 2.5 Hz and 3.5 Hz with no offset set at 1 V

**Figure 10:**
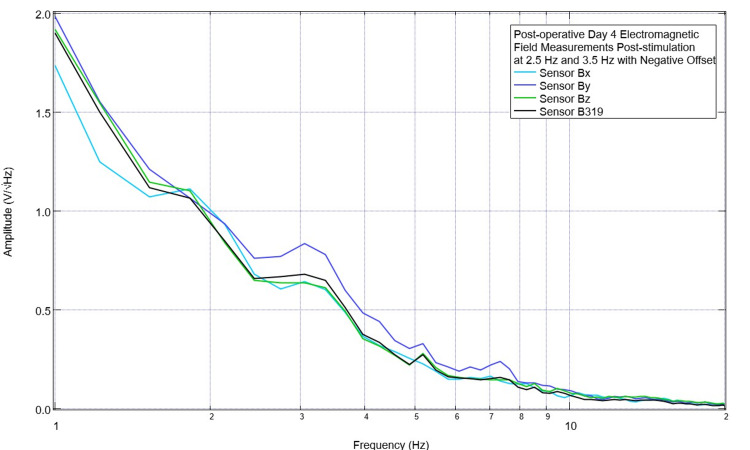
Swine electromagnetic field recordings on postoperative day 4 of electromagnetic field measurements from cortical circuits when stimulating with a negative offset of 500 mV Electromagnetic field recordings of the swine subject were obtained after using a negative offset of 500 mV. Otherwise, stimulation settings remained stable at 1 V. Stimulation occurred at 2.5 Hz and 3.5 Hz for two minutes each

**Figure 11:**
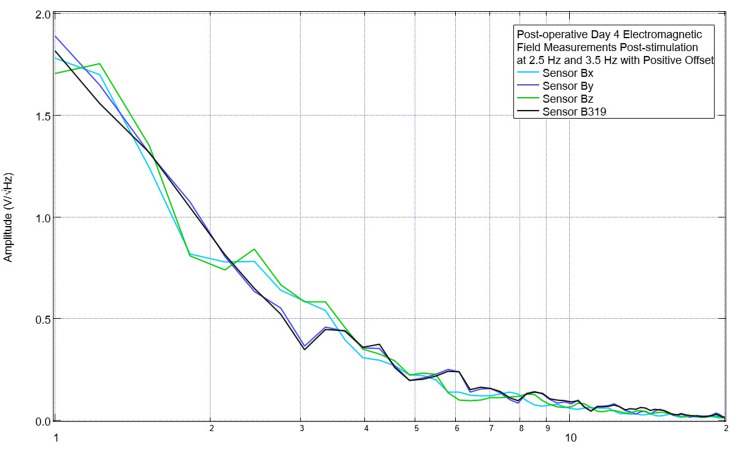
Evaluation of electromagnetic field measurements of the subject on postoperative day 4 after stimulation with a positive offset of 500 mV Electromagnetic field recordings of the swine subject were obtained after using a positive offset of 500 mV. Otherwise, stimulation settings remained stable at 1 V. Stimulation occurred at 2.5 Hz and 3.5 Hz for two minutes each

Due to these initial findings with morphologic changes at 2.5 Hz and 3.5 Hz after stimulation, additional investigations were done to evaluate the effects of using an offset in the EMF signal generator. This was done to shift the transmitted EMF sine wave to keep all transmissions positive and all negative. Assessments of post-stimulation EMF measurements of the subject occurred with no offset, a positive 500 mV offset, and a negative 500 mV offset. These are shown in Figures 9-11. With no offset, it was noted that there was a prominent peak at approximately 2.2 Hz measured in all sensors, while at 3.5 Hz, there was no noted peak. With an EMF stimulating negative offset at 2.5 Hz, all sensors demonstrated a valley and a peak at 3.1 Hz. With a positive offset, sensors Bx and Bz returned to having a peak at 2.5 Hz and at 3.5 Hz (+/- 0.2 Hz); these peaks were demonstrated in all sensors. Due to the changes in the positive direction at 3.5 Hz and 2.5 Hz with a positive offset, it was determined to continue to stimulate with a positive offset for the remainder of the trials.

**Figure 12:**
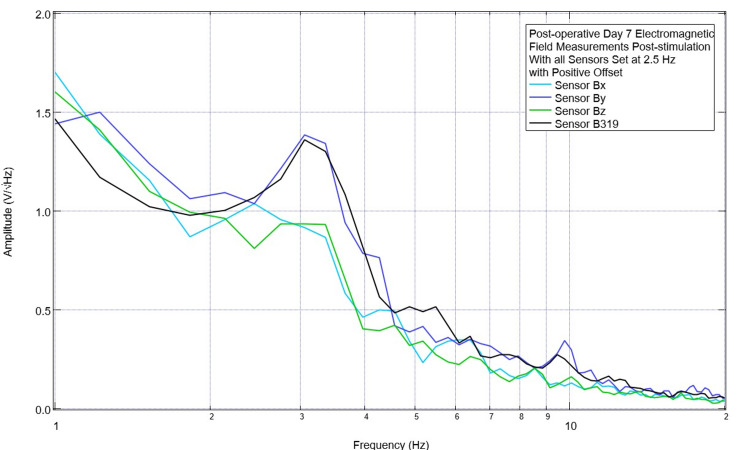
Post-stimulation measurements of the neural electromagnetic field on postoperative day 7 after stimulation with all sensors Post-stimulation measurements of the neural electromagnetic field on postoperative day 7 after stimulation at 2.5Hz with a positive 500 mV offset at 1 V when using all sensors simultaneously to stimulate; there is a large peak in amplitude at 3.1 Hz

In addition to stimulating with a positive offset, the decision was made to focus on stimulating at a singular frequency. As changes were more readily observed when stimulating at 2.5 Hz and there were changes noted postoperatively compared to preoperatively at 2.5 Hz, it was determined that continued trials would focus on stimulation at 2.5 Hz with a +500 mV offset set at 1 V. On the first day of these stimulation settings, it was decided to assess for changes that would occur if all sensors were used for stimulation simultaneously. These findings are shown in Figure 12. There was a large peak seen at 3.1 Hz, which had not been seen in previous trials. It was also noted that a downward slope at 2.5 Hz became a positive slope in sensor B319, a peak was formed in sensor Bx at 2.5 Hz when the initial slope was negative, and slopes became less negative in sensors By and Bz with positive slopes occurring just after 2.5 Hz post-stimulation. Additionally, the amplitudes were noted to be increased after stimulation compared to pre-stimulation with amplitudes noted to be less than 0.8 V/√Hz in all sensors prior to stimulation, with less than 0.5 V/√Hz in sensors Bz and less than 0.6 V/√Hz in Bx.

**Figure 13:**
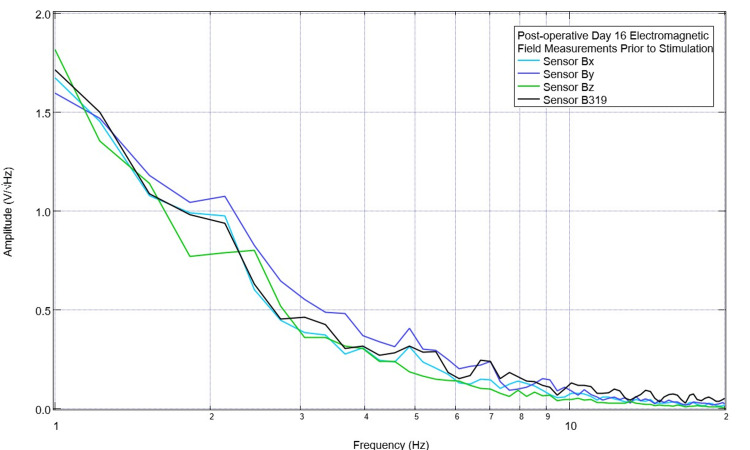
Pre-stimulation baseline measurements of the Yucatan miniswine obtained on postoperative day 16 Baseline recordings were obtained of swine cortical activity measured by the electromagnetic field sensors prior to stimulation on postoperative day 16

**Figure 14:**
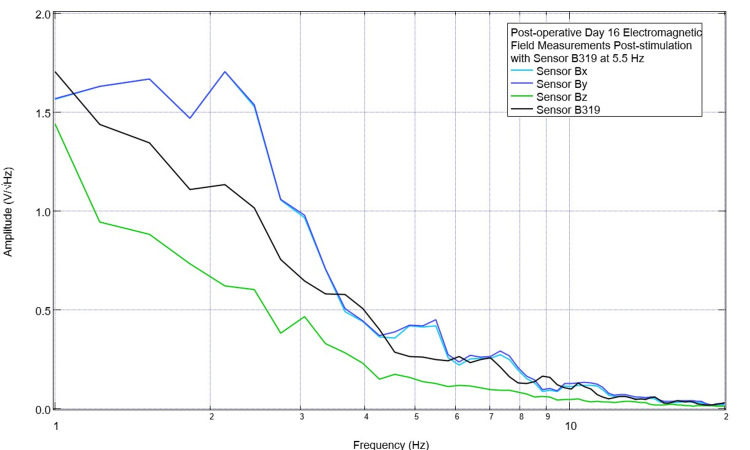
Post-stimulation electromagnetic field measurements after stimulating at 5.5 Hz with a positive 500 mV offset at 1 V on postoperative day 16 Electromagnetic field measurements were obtained by evaluating the cortically generated electromagnetic fields after stimulating at 5.5 Hz using a 500 mV positive offset at 1 V. Morphology differed compared to pre-stimulation evaluation

For further studies, it was determined to attempt to better target the EMF transmission and use a singular stimulator over time. Therefore, it was determined to continue to stimulate with a positive offset at 2.5 Hz using sensor B319 as the transmitting sensor with daily assessments of pre-stimulation and post-stimulation changes. This continued until postoperative day 16 when there was improvement at 2.5 Hz and 3.5 Hz. Still, there were valleys that had not yet recovered to baseline at 4.3 Hz and 5.5 Hz. Therefore, stimulation settings were changed to 5.5 Hz with a positive 500 mV offset at 1 V. Pre-stimulation EMF is shown in Figure 13. A valley was noted at 4.3 Hz and a small peak at 5.5 Hz in B319 and By but not in Bx or Bz. Post-stimulation measurements are shown in Figure 14 where, at 5.5 Hz, there were now peaks in Bx and By.

**Figure 15:**
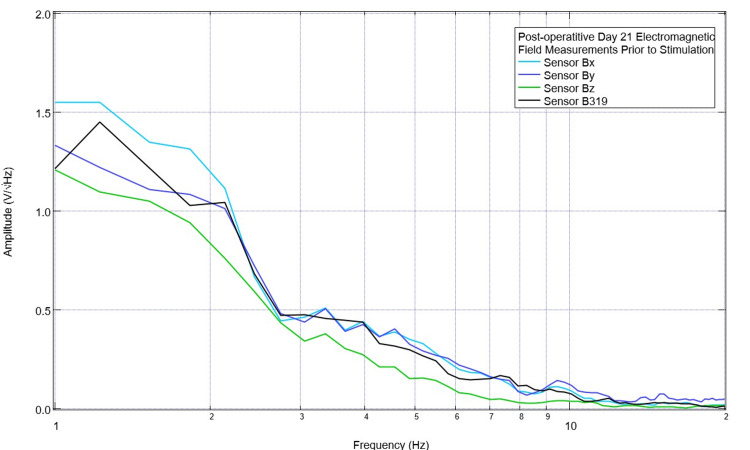
Final baseline, pre-stimulation electromagnetic field measurements of the swine obtained on postoperative day 21 Final pre-stimulation recordings of electromagnetic field measurements of the swine subject were completed on postoperative day 21

**Figure 16:**
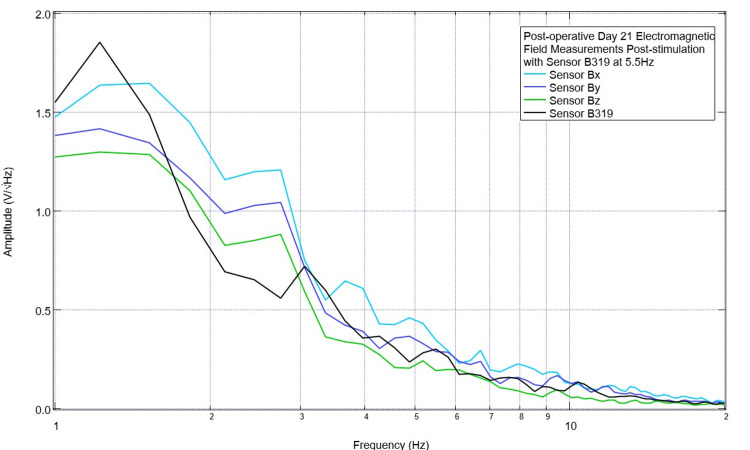
Final post-stimulation recording of neural electromagnetic fields from the swine on postoperative day 21 Final post-stimulation recordings of neural electromagnetic fields on postoperative day 21 were recorded from the swine, obtained after stimulation for 3 minutes set at 5.5 Hz with 500 mV positive offset at 1 V

Continued stimulation occurred at 5.5 Hz until postoperative day 21. On postoperative day 21, the final day of stimulation occurred with baseline preoperative and postoperative measurements. The pig was then euthanized on postoperative day 22 for future studies of serology and histology on the effects of this TBI model and stimulation on TBI. These final measurements are shown in Figures 15-16. As shown in Figure 15, there was a small peak noted at 5.5 Hz pre-stimulation, and at 4.5 Hz, there was a peak seen in sensors Bx, By, and Bz with a slightly negative slope seen in sensor B319. Post-stimulation, as shown in Figure 16, there was a peak at 4.5 Hz in sensor By, and a much more positive peak at 5.5 Hz in sensor B319; otherwise, the amplitudes were negative post-stimulation in the other sensors.

The preoperative, postoperative, and post-stimulation data sets evaluating the peaks and valleys in sensor B319 were queried using the AI LLM model. Reported data were assessed for hallucinations and common values that occurred in three or more measurements, or in the postoperative data set for more than 50% of readings. Using this model, it was noted that within the peaks in the preoperative data set, there was a more gradual increase in frequency between the frequency values of peaks compared to the postoperative data set which had increases in spacing between peaks. It was additionally noted by the AI system that the post-stimulation data set had similarities to both preoperative data sets and postoperative data sets, with more evenly distributed peaks as in the preoperative data, while other sequences noted increased spacing. When queried further, the model noted that the patterns in the post-stimulation data set were closer to those of the preoperative data set as stimulation trials progressed, with the sequences beginning on stimulation day seven having good similarity (postoperative day 17) and sequences beginning on and after stimulation day eight (postoperative day 20) having the best similarity. The AI model also reported that the postoperative data set had distinctly different patterns than the preoperative patterns regarding peaks. Within the preoperative data set, the subset of numbers occurring frequently in peaks were noted to be 2.5 Hz, 3.5 Hz, 5.5 Hz, 7.9 Hz, and 11 Hz. Within the postoperative data set the frequent peaks were noted to be 2.2 Hz, 4.2 Hz, and 9.2 Hz. Within the post-stimulation data set, the most frequently occurring peaks were 1.4 Hz, 2.2 Hz, 2.6 Hz, 5.2 Hz, 5.5 Hz, 7.5 Hz, 8.2 Hz, 8.8 Hz, and 10 Hz.

When evaluating the valleys, the AI model noted that the postoperative data set did not have identified patterns that resembled those in the preoperative or the post-stimulation data sets and were determined to be unique. In the evaluation of the preoperative data set, the model stated that the frequency values of the valleys tended to generally show a gradual increase between inflection points leading to a more evenly spaced graph with less variability. Similarly, within the peaks, there were larger intervals between valleys in the postoperative data set compared to the preoperative and post-stimulation data sets. The post-stimulation data set showed variability with some regions of more regular valleys during a selected day of measurement while other days of measurement showed more irregular patterns. This variability was not noted to be temporally associated with increased days of stimulation. The most frequently reported valleys in the preoperative data set were noted to be 2.2 Hz, 3.1 Hz, and 7.5 Hz. Within the postoperative data set, the most frequently reported valleys were 1.8 Hz, 3.9 Hz, 5.5 Hz, and 8.8 Hz. Finally, the most frequent valleys in the post-stimulation data set were 1.8 Hz, 2.5 Hz, 3.4 Hz, 7.2 Hz, 9.5 Hz, and 11 Hz. It was noted that there were some specific frequencies with overlap within the peaks and valleys as there were some measurements that occurred as both a peak and valley on differing days within the same category of preoperative, postoperative, and post-stimulation; however, to control for these occurrences, the most frequently appearing distinction was reported for these points. These points included 11 Hz, 9.2 Hz, and 7.5 Hz in the preoperative data set and 7.5 Hz in the post-stimulation data set.

## Discussion

EMFs have been found to be generated through electrochemical signaling at the neuronal level. These EMFs have been evaluated in a variety of different conditions [1,2,12,13,22]. Furthermore, TMS has been investigated in early-phase research as a treatment for brain injury, resulting in some early success in preclinical models with the reversal of histological injury and improvement in physiologic functioning [11,24]. In our investigations, we assessed whether it would be feasible to utilize a Yucatan minipig model of TBI to evaluate EMF in real-time in a non-invasive fashion through pre-CCI and post-CCI evaluations and targeted non-invasive EMF stimulation using induction sensors, an EMF signal generator, and an engineered EMF shielded helmet constructed with Mu-metal and copper mesh with EMF channels. We also aimed to assess the efficacy of stimulation on alterations in EMF and whether these alterations would restore the EMF patterns to baseline or if the EMF patterns in neuronal circuits would become closer to baseline.

In the evaluation of our primary objective, it was found that the method of inducing TBI using a gravity-dependent CCI model with preoperative and postoperative EMF measurements was feasible. Additionally, stimulation based on comparisons of preoperative and postoperative EMF evaluations was also feasible as appropriate measurements were able to be taken and the EMF could be effectively measured in real-time. As depicted in Figures 6-7, these measurements were effectively evaluated, and using licorice as a distraction allowed for appropriate attention from the pig to maintain its focus long enough to undergo the three-minute stimulation trials with measurements during these trials. Measurements were able to be completed preoperatively for seven measurements on seven separate days and through a 21-day postoperative period, thereby identifying that this methodology can be effectively used over long periods of time. This will enable potential further studies with larger applications and larger sample sizes by evaluating generated EMF in swine models and in the evaluation of the treatment of neuropathologic conditions in swine as a preclinical model for neurologic disease and treatment.

In the evaluation of our secondary aims, after identifying the techniques of evaluating EMF preoperatively and postoperatively with targeted stimulation based on differences between preoperative and postoperative measurements, we analyzed the efficacy of guided stimulation in a swine model. Using an AI LLM model to evaluate patterns within preoperative, postoperative, and post-stimulation measurements of peaks and valleys, we were able to determine that there are differences between preoperative, postoperative, and post-stimulation measurements of EMF as recorded in real-time through a non-invasive method. Similarly, it was noted that there was variation in the spacing of peaks and valleys within EMF measurements in both peaks in valleys in the postoperative assessments. This is compared to more organized, more evenly spaced peaks and valleys seen in the preoperative recordings and the more variable measurements with both evenly spaced and dispersed patterns of peaks and valleys seen post-stimulation. This is likely due to the disruption of cortical circuitry and changes in firing patterns within post-TBI swine. The CCI resulted in cortical and neuronal disruption causing disorders in signaling and action potential generation altering the ability to perform effects of spatial and temporal summation required for neuronal activity. These changes disrupted the normal cortical architecture and electrical and chemical signaling patterns resulting in changes in generated EMF as measured by this system. Likely, in the non-pathologic state, the more gradual spacing between peaks and valleys is from regular patterns of signaling while the injury disrupted the ability of the brain to participate in its regular functionality. As within the post-stimulation readings, the measurements varied between both the preoperative and postoperative assessments; the more disordered measurements may be those more closely related to injury while the more regular peaks and valleys may be due to improvements in cortical firing from healing. As the AI model noted regression of the EMF patterns in the peaks towards preoperative patterns with stimulation on stimulation days seven and eight with frequent stimulation, there may be some restoration of function at the circuit level with non-invasive stimulation in injured states. This would correlate with trials of TMS in rodent models where histological and physiologic recovery was noted with TMS [11].

Variability was noted in the effects of stimulation in post-stimulation measurements on the day of stimulation with some trials noting immediate recovery with a peak forming post-stimulation but others with a valley. This phenomenon will need to be further investigated in future studies. These effects may be due to hyperpolarization from stimulation causing the neuron to effectively be stunned in cases where the EMF measurement becomes a valley depressing the amplitude as it is unable to signal. However, due to the overall noted improvement in patterns with stimulation towards preoperative patterns, it may be seen that this immediate depression may be seen in a brief phase immediately post-stimulation and may not reflect the overall long-term changes on the neuron. In fact, due to the improvements towards the baseline note in the post-stimulation data set assessed by the AI (taken before the daily stimulation trial each day to evaluate the durable effects of stimulation), this depression may allow for a “resetting” of the neuron such that it can then resume its baseline patterns of firing similar to the treatment effects of defibrillation/cardioversion in cardiac myocytes. This has been supported by some studies noting depolarizations and alterations in excitability post-stimulation [25]. Additionally, from a histological perspective, the return to baseline EMF signaling is thought to represent the preservation or regeneration of neural function. Necessarily, this would require less apoptosis within the region of injury or the promotion of healing through the effects of stimulation. Further studies are required on the cellular level to evaluate this hypothesis. A summary of selected observations with theorized physiologic explanations is presented in Table 1.

**Table 1:** Overall patterns identified in EMF measurements with theorized physiologic explanations EMF: electromagnetic field

Category	Finding	Explanation
Postoperative peaks	Increased spacing between peaks compared to the preoperative scenario	Impaired neural function with decreased spatial and temporal summation
Postoperative peaks	Decreased variability in changes in slope between peaks	Impaired neural function with decreased spatial and temporal summation
Postoperative peaks	AI identified differences in patterns compared to the preoperative scenario	Altered neural circuitry causing changes in generated electromagnetic fields
Post-stimulation peaks	More evenly spaced peaks consistent with patterns seen preoperatively	Preservation or regeneration of neural circuits due to reduced apoptosis or neuroplasticity
Post-stimulation peaks	AI identified similarity in overall pattern compared to preoperative patterns beginning postoperative day 20	Preservation or regeneration of neural circuits due to reduced apoptosis or neuroplasticity
Post-stimulation peaks	AI identified the distinction between patterns in post-stimulation data and postoperative data	Altered neural circuitry causing changes in generated electromagnetic fields
Postoperative valleys	Increased spacing in between valleys compared to the preoperative scenario	Impaired neural function with decreased spatial and temporal summation
Post-stimulation valleys	Variability in spacing with some valleys more regularly spaced while other days with increased spacing	Impaired neural function with decreased spatial and temporal summation with signs of recovery

These findings represent changes in preoperative from postoperative EMF measurements. This appears to confirm similar findings demonstrated in previous studies that these sensors, helmet, and EMF channel construct can effectively measure and identify differences in preoperative and postoperative EMF generated by cortical neurons [9]. The observed values within peaks and valleys preoperatively and postoperatively did appear to have variation compared to the previously published values [9]; as an example, the 2.5 Hz peak preoperatively in this data set, which was a postoperative peak as previously reported, and the frequent 6.5 Hz peak in this trial and previous study [9]. This difference may be due to unique firing patterns of neural circuits within each pig. Despite these differences, there were frequencies with consistencies seen compared to previous studies. There was an overlap between this investigation and a previous investigation of swine EMF with an 11 Hz valley seen postoperatively in previously published work and as a valley in the post-stimulation data set in this trial [9]. Similarly, there was a large interval between the peaks in the postoperative data set, which compares favorably to the identified trend in a previous study evaluating postoperative changes with fewer inflection points noted between 4.5 Hz and 7.5 Hz [9].

In evaluating the post-stimulation EMF, the AI model noted that the patterns within the post-stimulation EMF were overall different when comparing the preoperative and postoperative data. Of note, the AI model noted that the patterns within the post-stimulation data sets appeared to resemble the preoperative data sets beginning on the seventh day of stimulation (postoperative day 17). This may be secondary to the effects of treatment as, in previously identified studies, the postoperative EMF patterns were not reported to have converged towards the preoperative measurements regardless of temporal profile [9]. The variability in certain patterns of specific points will need to be further investigated with frequencies such as 11 Hz, 7.5 Hz, and 9.2 Hz appearing as both a peak and a valley in multiple data sets.

In initial assessments, the EMF signal generator did not utilize an offset of the signal. It was found that using a positive offset of 500 mV was more effective than the 0 mV offset for a 1 V-shaped sinusoidal stimulation when targeting the treatment of peaks. This is seen in Figure 11 with a recovery of 2.5 Hz from a valley. Using a positive offset with all sensors set at 2.5 Hz created a large peak occurring at 3.1 Hz with an increase in slope at 2.5 Hz, as seen in Figure 12. This may be due to the location of the center of the sinusoidal wave. Without an offset, the 1 V sinusoidal waves have a maximum amplitude of 1 V centered around 0 V. Therefore, the maximum and minimal amplitudes of the wave are -500 mV and 500 mV. When a 500 mV positive offset was set, the bottom of the sinusoidal wave is 0 mV with a maximum of the sinusoidal wave now being located at 1 V with the center of the wave at the positive 500 mV offset. This keeps the entire EMF generated within positive amplitudes when transmitted to the subject. In future studies, evaluation of the stimulation in the negative direction could be done in order to assess the loss of common valleys from preoperative to postoperative data sets, and it is proposed that in these studies, it may be best suited to utilize a negative offset to keep the stimulation threshold negative.

Limitations

There are several limitations to this study. Primarily, this study was conducted with a small sample size, which may limit the generalizability of its findings. Further studies are needed to evaluate the efficacy of this modality in the treatment of TBI in additional preclinical models of TBI and in more swine. Additionally, further behavioral data is needed to empirically evaluate the effects of stimulation on post-TBI recovery. An additional limitation is due to the AI LLM Model. LLM models have limitations in the evaluation of data with a potential for the development of AI “hallucinations”; further investigations that utilize larger samples with unsupervised learning models may be more efficacious in evaluating EMF patterns to develop additional models for effect. Finally, as the EMF signal generator has numerous functions and variables including factors such as altered transmitted signal shape, increased amplitudes of signals, and differing offsets and frequencies, there may be additional settings and offsets that may be more efficacious in the treatment of the disease process other than the ones measured here. Moreover, initial craniotomy effects may also cause changes in EMF measurements, which are yet to be explored; however, this would appear to be less likely as stimulation resulted in EMF recovery that would not effectively treat such a breach effect and is, therefore, more likely to be caused by the direct cortical injury. Lastly, as this study utilized a gravity-controlled method of CCI, there may be a degree of variability with cortical injury compared to prior studies. Additional studies with continued evaluation of effects on swine models including investigation of histology and biomarkers in addition to effects on EMF are needed to evaluate these potential variables.

## Conclusions

Based on our findings, measuring neuronal circuits within a swine model of TBI is feasible using a Mu-metal and interlaced copper mesh shielded helmet, EMF channels, and induction sensors. Furthermore, it has been observed that it is possible to use stimulating technologies on the EMF abnormalities in neuronal circuits created by TBI. There appear to be changes in baseline EMFs post-TBI and with stimulation, and AI modeling has noted that patterns in post-stimulation neuronal activity trend towards being consistent with preoperative baseline patterns. These findings suggest that real-time non-invasive measurements with this system may help guide EMF stimulation technology in the correction of EMF signal abnormalities for the potential treatment of cortical injury. Further studies are needed to evaluate the clinical significance of these patterns and to confirm their reliability and reproducibility.
